# Case Report, Practices Survey and Literature Review of an Under-Recognized Pediatric Vascular Disorder: The BASCULE Syndrome

**DOI:** 10.3389/fped.2022.849914

**Published:** 2022-04-07

**Authors:** Natacha Baurens, Clémence Briand, Lisa Giovannini-Chami, Jean-Marie De Guillebon De Resnes, Thomas Hubiche, Christine Chiaverini, Pascal Giordana, Georges Leftheriotis, Julie Bernardor

**Affiliations:** ^1^CHU Lenval, Hôpitaux Pédiatriques de Nice, Nice, France; ^2^Unité de Dermatologie, Hôpital Archet-2, CHU de Nice, Nice, France; ^3^Université Côte d’Azur, Faculté de Médecine, Nice, France; ^4^Unité de Néphrologie et de Rhumatologie Pédiatrique, Hôpital L’Archet, CHU de Nice, Nice, France; ^5^Unité de Médecine Vasculaire, Université Côte d’Azur, CHU Nice, Nice, France; ^6^Unité de Médecine et d’Explorations Vasculaires, CHU Pasteur et CHU Pédiatrique Lenval, Nice, France

**Keywords:** acrosyndrome, children, BASCULE syndrome, orthostatic intolerance, POTS

## Abstract

**Introduction:**

Bier anemic spots, cyanosis, and urticaria-like eruption (BASCULE) syndrome is an underreported pediatric vascular disorder from the group of acrosyndromes. In children, these include paroxysmal acrosyndromes (Raynaud’s phenomenon and chilblain-like lesions), permanent acrosyndromes (acrocyanosis), and transient acrosyndromes, in which their pathogeneses are associated with virus infections, Epstein-Barr virus, and, more recently, SARS-CoV-2, respectively.

**Methods:**

We reported a case of BASCULE syndrome associated with postural orthostatic tachycardia syndrome (POTS) and provided a narrative review of case reports describing the BASCULE syndrome in children. Moreover, we presented the results of a prospective practice survey that we performed in the French medical community.

**Results:**

A 14-years-old boy reported pruritic erythrocyanic lesions on the lower limbs, which occurred whenever he was in a standing position and fully resolved when he laid down. He reported asthenia and cramps. He presented a typical BASCULE syndrome associated with POTS confirmed by a tilt-test. Physical and vascular examinations were within the normal range. We identified 12 case reports, describing 21 pediatric cases since 2016. Most patients were adolescents between 12 and 19 years of age or were newborns. Furthermore, 20% of cases in the literature have presented POTS or orthostatic intolerance. Our survey among 95 French physicians confirmed that BASCULE syndrome is an underdiagnosed and under recognized disease in the general pediatric practice, at least in France. Among these physicians, 65% had already encountered patients with similar symptoms, but only 30% declared that they had knowledge of the BASCULE syndrome.

**Conclusion:**

The under-recognition of the clinical manifestations leads the patients to consult emergency rooms, with multiple unnecessary investigations performed. Therefore, we suggest that the diagnosis of BASCULE syndrome is based on clinical observations, without the need for laboratory tests, to avoid unnecessary health costs. We suggest physicians to perform a tilt-test when POTS is suspected.

## Introduction

Bier anemic spots, cyanosis, and urticaria-like eruption (BASCULE) syndrome is an underreported pediatric vascular disorder from the group of acrosyndromes. In children, these include paroxysmal acrosyndromes (Raynaud’s phenomenon and chilblain-like lesions), permanent acrosyndromes (acrocyanosis), and transient acrosyndromes.

The “BASCULE” syndrome has been first described in 2016 by Bessis et al. (1) in four children as a dermatosis associated with bier anemic spots, cyanosis, and urticaria-like eruption.

The symptoms mostly affect the lower limbs, sometimes the forearm, and are generally associated with tenderness, itching, and edema. Painful sensations have been reported by some patients. The clinical manifestation is induced in a standing position or by manual compression and is fully reversible when lying down or walking (2, 3). In some cases, BASCULE syndrome has been associated with postural orthostatic tachycardia syndrome (POTS) and other forms of orthostatic intolerance (OI), with conditions including dizziness, palpitations, tremulousness, and leg weakness detectable in the upright position (4, 5). POTS syndrome is caused by cerebral hypoperfusion, in which the symptoms include lightheadedness, syncope, palpitations, and fatigue. It is associated with an increase in heart rate to >30 bpm (>40 bpm in children/adolescents younger than 19 years) within 10 min of standing (or during a head-up tilt-test to at least 60°) in the absence of orthostatic hypotension (6).

To date, the frequency and prevalence of the BASCULE syndrome in the general population are unknown.

We reported a typical case of BASCULE syndrome associated with POTS, followed by a review of the available literature on this under-recognized syndrome. Moreover, we conducted a French national prospective practice survey about BASCULE syndrome, involving dermatologists, pediatric rheumatologists, and vascular physicians.

## Materials and Methods

### Literature Review

**FIGURE 1:**
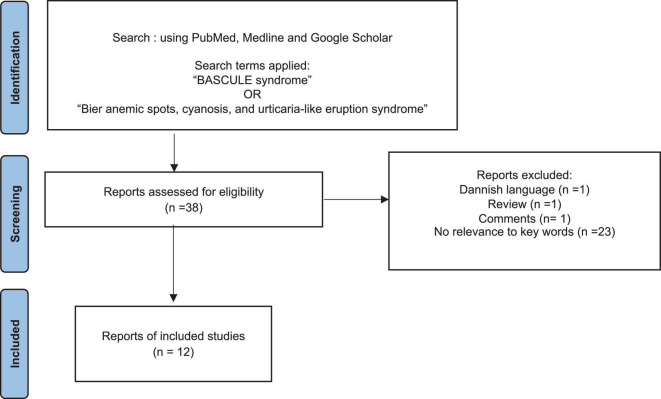
Study selection flowchart. We conducted a literature review using the terms “BASCULE syndrome” OR “Bier Anemic spots, cyanosis, and urticaria-like eruption syndrome” in PubMed, Medline, and Google Scholar databases. The process of study selection is synthesized. The causes of exclusion at each step are reported (the number of studies excluded for each individual cause). Of the 38 publications retrieved by the literature search, 12 were included.

For a systematic review of the literature, we used PubMed, Medline, and Google Scholar search engines for articles containing terms such as “BASCULE syndrome” OR “Bier Anemic spots, cyanosis, and urticaria-like eruption syndrome.” Among the 38 publications retrieved by the literature search, we included those articles reporting on case series or case reports in English language, and 12 were selected for further reading based on the title and abstract. Studies with patients older than 21 years of age, as well as reviews or articles without relevance to keywords, were excluded. Data for clinical, POTS evaluation, and treatment were collected and summarized in Table 1. We created a Preferred Reporting Items for Systematic Reviews (PRISMA) flowchart showing the results of the literature search (Figure 1).

**TABLE 1:** Bibliometric analysis of bier anemic spots, cyanosis, and urticaria-like eruption (BASCULE) syndrome cases.

References	Article	Cases	Age	Sex	POTS/OI	Treatment
Bessis et al. (1)	Case report (letter to the editors)	4	3 months	F	POTS (1/4)	None
			14 years	F		Desloratadine 10 mg/day for 7 days (effective on pruritus)
			15 years	M		H1-antihistamine and tranexamic acid (ineffective)
			19 years	M		None
Bessis et al. (4)	Case report (letter to the editors)	2	3 months	M	No	None
			16 years	F		None
Jiménez-Gallo et al. (11)	Case report	1	8 years	M	No	H1-antihistamine (ineffective)
Danescu et al. (12)	Case report	1	11 years	F	OI	None
Martín et al. (5)	Case report	1	13 years	F	OI	None
Barbé et al. (13)	Case report (brief report)	2	6 months	NA	No	None
			5 months			None
El Nemnom et al. (2)	Literature review and case report	2	13 years	F	No	Desloratadine 10 mg/day for 6 weeks (ineffective)
			13 years	F		
						None
Piroth et al. (3)	Case report (letters to the Editor)	2	5 months	NA	No	None
			8 months			None
Cunningham et al. (8)	Case report	1	16 years	M	No	Cetirizine 10 mg/day & Bilastine 20 mg/day and Aspirine (ineffective) then Bilastine 80 mg/day (complete remission but recurrence when dose reduction)
Ramírez-Lluch et al. (14)	Case reports (letter to the editors)	2	13 years	M	No	None
			13 years	M		
Guillen-Climent et al. (9)	Case report	1	13 years	F	OI	Bilastine 40 mg/day (effective on pruritus)
Berrebi et al. (15)	Case report	1	10 years	M	No	No

*BASCULE, Bier anemic spots, cyanosis, and urticaria-like eruption; M, male; F, Female; POTS, Postural Orthostatic Tachycardia Syndrome; OI, orthostatic intolerance; NA, not available.*

### Medical Survey

To determine the frequency and the knowledge about the BASCULE syndrome in the medical pediatric community, we conducted a web-based survey among dermatologists, rheumatologists, and vascular physicians (French Society of Pediatric Dermatology, French Society of Pediatric Rheumatology, and French Society of Vascular Medicine). The questionnaire had three sections with twenty close-ended questions with an additional free-form text field (Supplementary Material 1). The first section was about the physician’s characteristics, such as specialty and way of practicing. The second section was focused on observation and experience concerning acrosyndrome in children. The third section of the questionnaire was designed to assess the physicians’ knowledge and practices about BASCULE syndrome.

## Case Presentation

A 14−year−old boy had been reporting pruritic erythrocyanic lesions on the lower limbs for approximately 5 months. Both lower limbs were affected, and symptoms were presented in an ascending mode and occurred systematically in a standing position. Similar symptoms were observed for the hands, but these only appeared sporadically. The patient reported a feeling of asthenia and cramps. All symptoms disappeared when he lay down or was walking. No edema had been detected.

The symptomatology was initially associated with an episode of cough and rhinorrhea during the COVID-19 pandemic, although the SARS-CoV-2 PCR and serology were negative. No other medical history or other virus infection-associated symptoms, such as fever, dyspnea, or arthralgia, were found. The family anamnesis showed neurocardiogenic (i.e., “vasovagal”) syncope present in the patient’s father and grandmother from the father’s side.

**FIGURE 2:**
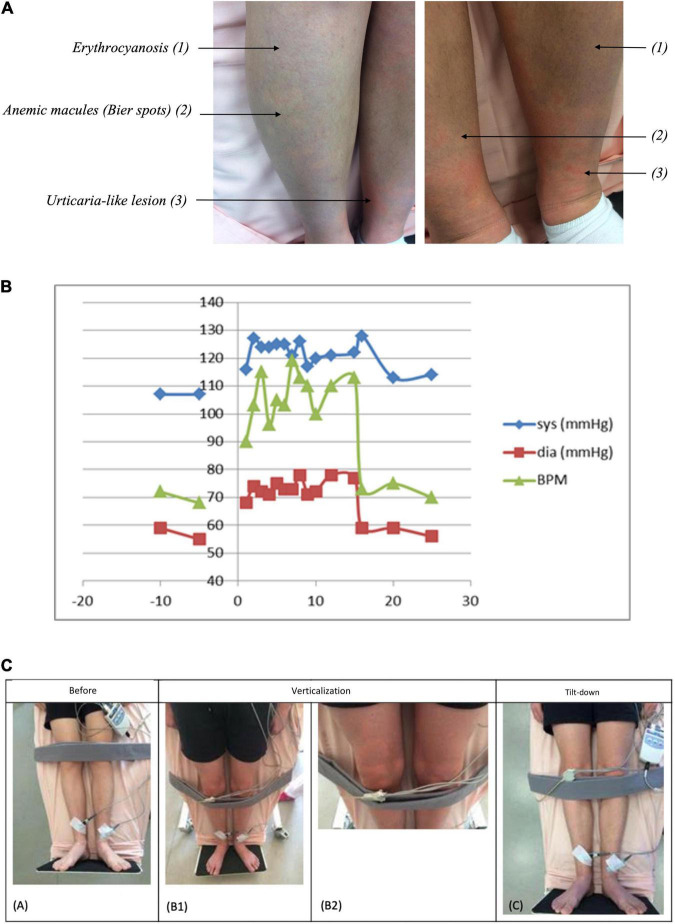
**(A)** Bier Anemic spots, cyanosis, and urticaria-like eruption (BASCULE) of lower limbs while orthostatism in a 14-year-old patient. Skin eruption is characterized by erythrocyanic background (1) with anemic macules (Bier spots) (2), and urticaria-like eruption (3). **(B)** Blood pressure and heart rate evolution during tilt-test. The blue curve represents the systolic pressure, the red curve represents the diastolic pressure, and the green curve represents the average heart rate as a function of time before and after tilt-test. After verticalisation, heart rate increased to 110–120 bpm while blood pressure remained around 120/70 mmHg, which is characteristic of a postural orthostatic tachycardia syndrome (POTS) associated with cerebral hypoperfusion–symptoms include lightheadedness, syncope, palpitations, and fatigue. This condition is associated with an increase in heart rate >30 bpm (or >40 bpm in children/adolescents younger than 19 years) within 10 min of standing (or during a head-up tilt-test to at least 60°) in the absence of orthostatic hypotension. **(C)** Clinical course during the tilt-test. Clinical features before the tilt-test **(A)**, during the tilt-test **(B1,B2)**, and after the tilt-test **(C)**.

Physical examinations, including biometry, and vascular examinations (pulse), were within the normal range. However, 10 min of orthostatism triggered a skin eruption characterized by anemic macules on an erythrocyanic background with hatching in the lower limbs (Figure 2A). All symptoms disappeared spontaneously when he returned to a supine position. Results from blood tests, including complete blood count, coagulation and renal and liver function parameters, inflammatory markers, and autoimmune disease screening, were within the normal range. No antinuclear antibodies, antiextractable nuclear antibodies, anticardiolipin antibodies, and cryoglobulins were detected. The thyroid function test did not suggest primary hypothyroidism or hyperthyroidism. Serum tryptase levels at 30 min and 1 h 30 min after orthostatism were unchanged (2.7 μg/l was the initial value, 2.84 μg/l after 30 min of orthostatism, and 2.52 μg/l after 1 h 30 min of orthostatism). Doppler ultrasound examinations of lower limbs and abdominal and pelvic areas were clinically unremarkable. Thoraco-abdomino-pelvic CT revealed two unspecific nodules on the upper-right lobe, but pelvic compression could be excluded.

Next, we performed a 75° head-upright tilt-test in order to explore POTS or OI. The resting ECG was in the normal range, heart rate was 70 bpm, and blood pressure was 110/60 mmHg. After verticalization, heart rate increased to 110–120 bpm, while blood pressure remained around 120/70 mmHg (Figure 2B). In the upright position, the patient has progressively developed bilateral cutaneous erythema extending from the feet to the thighs with hypopigmented macules (i.e., Bier’s spots) and urticaria-like areas associated with pruritus. The rash persisted throughout the upright test, affecting the lower limbs and the hands to a lower extent, without edema. All symptoms disappeared within 5 min after returning to the lying position (Figure 2C). The clinical symptoms appeared consistent with a diagnosis of BASCULE syndrome associated with POTS.

No specific treatment, such as physical exercise and dietary modifications (daily hydration >1.5 l/24 h with increased sodium intake), were recommended but without significant improvement. Anti-H1 antihistaminic (Bilastine 40 mg two times daily, so 2 mg/kg/day) treatment was introduced in addition to fludrocortisone (0.1 mg per day) to improve postural tachycardia. However, it was only effective after several months on itching sensation but not on other symptoms.

## Results

### Literature Review

A total of 21 pediatric cases have been reported since 2016 (Table 1). Most patients were adolescents between 12 and 19 years of age or newborns in the first few months of life. Bessis et al. (1) and Barbé et al. (7) have described three cases of infants with a “blue-white-red” rash on the legs presenting as unusual crying noticed by parents after they have carried their child for a few minutes. Among cases reported, one has presented prolonged QT interval and one patient had a first−degree atrioventricular block associated with OI, with no evidence of correlation with POTS. Furthermore, five patients were diagnosed with OI, of which two were with POTS, including our current case, and three were with OI without tachycardia (20% of cases in the literature).

### Medical Survey

Among the 95 physicians who replied to the survey, 65% had already encountered patients with similar symptoms, but only 30% declared that they had knowledge of the BASCULE syndrome (Supplementary Material 1). Additional investigations were carried out by the majority of the interviewed physicians, including blood tests in 70% of cases (auto-antibodies screening, hemogram, serum chemistry, and coagulation tests), capillaroscopy (51%), and ultrasound scanning of the affected limbs (49%). A skin biopsy was performed in 8% of cases only. These additional investigations were always negative, except for the tilt-test, which may highlight an associated POTS syndrome.

## Discussion

Our patient presented typical syndromes associated with the BASCULE syndrome. Symptoms of BASCULE syndrome usually appear in the lower limbs while standing or by manual compression and resolve by lying down or walking. Lesions can be associated with tenderness, pruritus, edema, and possible pain (3).

Pathophysiological mechanisms of this affection remain largely unknown. Venous pooling induced by orthostatism appears to be the trigger of an erythrocyanosis and anemic macules, while urticaria−like eruption could result from mast−cell−degranulation in response to hypoxia (2). In our case, the absence of increased seric tryptase levels during orthostatism does not support the hypothesis of systemic mast cell degranulation but does not rule out the possibility of a local response that is not detectable in the plasma sample. Studies performed on skin biopsies of the urticaria-like eruption reported dilated capillaries with sparse interstitial eosinophils, as well as non-specific lymphocytic and mast cell infiltrates (1, 3).

The frequency and prevalence of the BASCULE syndrome remain unknown. Our practices survey results confirm that BASCULE syndrome is likely underdiagnosed and under-recognized in the general pediatric practice, at least in France. Although the triggering mechanism seems reproducible, the cutaneous symptoms could mimic compression syndrome, coagulation disorders, vasculitis, thyroid, or autoimmune disease. The underrecognition of the clinical manifestations leads the patients to consult in an emergency room, with multiple investigations (blood tests, ultrasound imaging, skin biopsies, etc.), especially when the symptoms occurred in infants. Physicians should be more aware of BASCULE syndrome: the knowledge of this diagnostic could permit to avoid all examinations to decrease unnecessary health expenses. Indeed, these patients are systematically referred to specialists or emergency units with increased family anxiety and diagnostic delay. The course of BASCULE syndrome is unpredictable. Although this acrosyndrome is benign and often improves spontaneously, the symptoms affect the quality of life. Further studies need to be performed to improve our understanding of the underlying pathophysiological mechanisms and propose better therapeutic and symptomatic management in severe cases.

To date, there is no effective treatment for the BASCULE syndrome and no consensus on the clinical management was available, although the symptomatology seems to resolve spontaneously. A total of 81% of specialists of the survey offered only symptomatic pieces of advice, e.g., performing exercise and avoiding prolonged orthostatism. Based on the hypothesis, the causative mechanisms are the mast cell degranulation, and treatment with anti-H1 antihistaminics, such as desloratadine, cetirizine, or bilastine, has been proposed in the literature (8, 9). They were applied in 33% of patients reported in the literature (7 of 21 including our case) and by 11% of physicians of our survey but proved ineffective in most cases. As described in the present case report, only one report by Cunningham et al. (8) presented a case of a 16-year-old boy whose pruritus were successfully treated by high doses of bilastine (2 mg/kg/day); however, his symptoms relapsed at lower doses. Furthermore, POTS management is based on no specific treatment (such as increasing fluid intake to 2–3 L per day, increasing salt consumption, and exercises) and medications included benzodiazepines, beta-blockers, antidepressants, and vasoconstrictors (10).

## Conclusion

Bier anemic spots, cyanosis, and urticaria-like eruption syndrome is a frequent but under-recognized chronic dermatosis reported in childhood and adolescence. The pathophysiological mechanisms are still poorly understood and need further studies to examine the mechanism of a functional peripheral and/or local dysautonomia of the cutaneous microcirculation and of a local histamine release. To date, there is no effective treatment for the BASCULE syndrome, and no consensus on the clinical management was available. Although the symptomatology seems to resolve spontaneously, the long-lasting symptoms have a negative impact on the quality of life of the patients. Given the reproducible character of this syndrome and the frequent negative results of the examinations usually carried out, the diagnosis of the BASCULE syndrome is mostly based on clinical observations and there is no need for laboratory tests. We suggest performing at least a tilt-test when POTS is suspected.

## Data Availability Statement

The raw data supporting the conclusions of this article will be made available by the authors, without undue reservation.

## Ethics Statement

Written informed consent was obtained from the individual(s) for the publication of any potentially identifiable images or data included in this article.

## Author Contributions

NB and JB worked on the conception and the design of the manuscript. GL, LG-C, CB, TH, CC, and PG worked on conception and review of the data. All authors contributed to the article and approved the submitted version.

## Conflict of Interest

The authors declare that the research was conducted in the absence of any commercial or financial relationships that could be construed as a potential conflict of interest.

## Publisher’s Note

All claims expressed in this article are solely those of the authors and do not necessarily represent those of their affiliated organizations, or those of the publisher, the editors and the reviewers. Any product that may be evaluated in this article, or claim that may be made by its manufacturer, is not guaranteed or endorsed by the publisher.
