# Supplementary material for: Case Report, Practices Survey and Literature Review of an Under-Recognized Pediatric Vascular Disorder: The BASCULE Syndrome
Source: Front Pediatr. 2022 Apr 7;10:849914. doi: 10.3389/fped.2022.849914 (PMC9021422; doi:10.3389/fped.2022.849914)

Practice survey: Questions

1) What medical specialty do you practice?

- ☐ Pediatrics / Rheumatopediatrics / Dermatopediatrics
- ☐ Dermatology
- ☐ Vascular medicine
- ☐ Rheumatologist

2) What is your mode of exercise?

- ☐ Hospital
- ☐ Liberal`
- ☐ Mixed

3) Do you know the definition of an acrosyndrome?

- ☐ Yes
- ☐ No

PART 1: ACROSYNDROMES

A vascular acrosyndrome corresponds to a vasomotor disorder of the extremities.
There are several forms:

- cold acrosyndromes, which can be paroxysmal: essentially **Raynaud's phenomenon**
- or permanent: **acrocyanosis, acrorhigosis** and **chilblains.**

The main paroxysmal acrosyndrome secondary to the heat is **erythermalgia.**
Most of the time, acrosyndromes are benign, but there are secondary forms in the context of arteriopathy or systemic diseases that should not be overlooked.

4) Have you ever had children with an acrosyndrome among your patients?

- ☐ No
- ☐ < 1 year
- ☐ 1-4 years old
- ☐ 5-14 years old
- ☐ 15-18 years old

5) If yes, which one(s)?

- ☐ Raynaud's syndrome

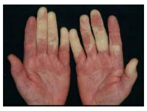

- ☐ Acrocyanosis

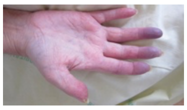

- ☐ Chilblain

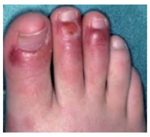

- ☐ Erythermalgia

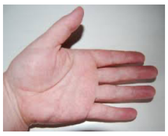

- ☐ Acroparesthesia

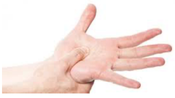

- ☐ Other

6) What is the proportion?

	None	< 5 cases	5 to 10 cases	>10 cases
**Raynaud's syndrome**				
**Acrocyanosis**				
**Chilblain**				
**Erythermalgia**				
**Acroparesthesia**				
**Other**				

7) Could you quantify the total number of cases of acrosyndromes encountered in children over the past 10 years?

- ☐ None
- ☐ 1 to 4 cases
- ☐ 5 to 9 cases
- ☐ 10 to 15 cases
- ☐ More than 10 cases

8) During which season are the most patients consulting you for this problem?

- ☐ Spring
- ☐ Summer
- ☐ Autumn
- ☐ Winter

9) Faced with a child's acrosyndrome (ie < 18 years old), do you systematically carry out additional examinations?

- ☐ Yes
- ☐ No
- ☐ Other (free text): ____________________________

10) What additional examinations have you already prescribed?

- ☐ Nail capillaroscopy
- ☐ Doppler echo
- ☐ Autoantibody test
- ☐ Cold agglutinins
- ☐ Skin biopsy
- ☐ Tilt test
- ☐ Other, give details: ____________________________

11) In the context of an acrosyndrome, have you found any family history?

- ☐ Systematically
- ☐ Often
- ☐ Rarely
- ☐ Never
- ☐ Comment: ____

12) Depending on your specialty, to whom do you refer these patients?

- ☐ Dermatologist
- ☐ Vascular physician (Angiologist)
- ☐ Rheumatologist
- ☐ Pediatrician
- ☐ Other: (specify) ____

13) What treatments do you prescribe?

- ☐ Physical measures (protection against cold/heat, heaters, etc.)
- ☐ Local treatments
- ☐ Calcium channel blockers
- ☐ Alpha-blockers
- ☐ Prostaglandins
- ☐ Other: (specify) ____

PART 2: BASCULE SYNDROME

14) Do you know the "BASCULE" syndrome?

- ☐ Yes
- ☐ No

The acronym BASCULE for " Bier's anemic spots cyanosis urticaria-like eruption" was described in 2016 [1] such as a new form of acquired, chronic and benign vasomotor dermatosis occurring electively on the lower limbs, in an orthostatic and immobile position, and characterized by:
(1) successive development of acrocyanosis, anemic macules, and pseudourticarial rash;
(2) the frequent presence of pruritus, tenderness or swelling of the affected limbs and
(3) rapid reversibility of all symptoms in clinostatism.

This impairment may be associated with a postural tachycardia syndrome (POTS) defined by an increase in heart rate greater than 30 beats per minute within 10 minutes of standing, without a drop in blood pressure. In children and adolescents, this elevation threshold is increased to 40 beats per minute.

[1] Bessis, D.; Jeziorski, É.; Rigau, V.; Pralong, P.; Pallure, V. Bier Anaemic Spots, Cyanosis with Urticaria-like Eruption (BASCULE) Syndrome: A New Entity? Br. J. Dermatol. 2016, 175 (1), 218–220.

15) Have you ever seen this type of lesion?

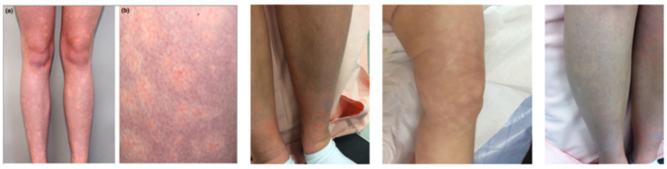

- ☐ Yes
- ☐ No

16) In what age group did you observe them?

- ☐ No observed
- ☐ < 1 year
- ☐ 1-4 years old

- ☐ 5-14 years old
- ☐ 15-18 years old

17) If yes, how many cases have you encountered in the past 10 years?

- ☐ ≤ 5 cases
- ☐ > 5 cases?

18) Faced with this syndrome, what additional examinations did you request?

- ☐ Nail capillaroscopy
- ☐ Doppler echo
- ☐ Autoantibody test
- ☐ Cold agglutinins
- ☐ Skin biopsy
- ☐ Tilt test
- ☐ Other, give details : ____

19) What treatments did you prescribe?

- ☐ Symptomatic measures: physical exercise, elevation of the legs
- ☐ calcium channel blocker
- ☐ Aspirin
- ☐ H1 antihistamines
- ☐ Other, give details : ____

20) What was the evolution?

- ☐ Spontaneously favorable
- ☐ Favorable under treatment
- ☐ Persistence of symptoms
- ☐ Worsening of symptoms
- ☐ Appearance of another associated pathology

Free comment:

City of practice (postal code): ____________

Thank you for your participation

Natacha BAURENS, Pediatrics fellow (CHU Nice)

Practice survey: Results

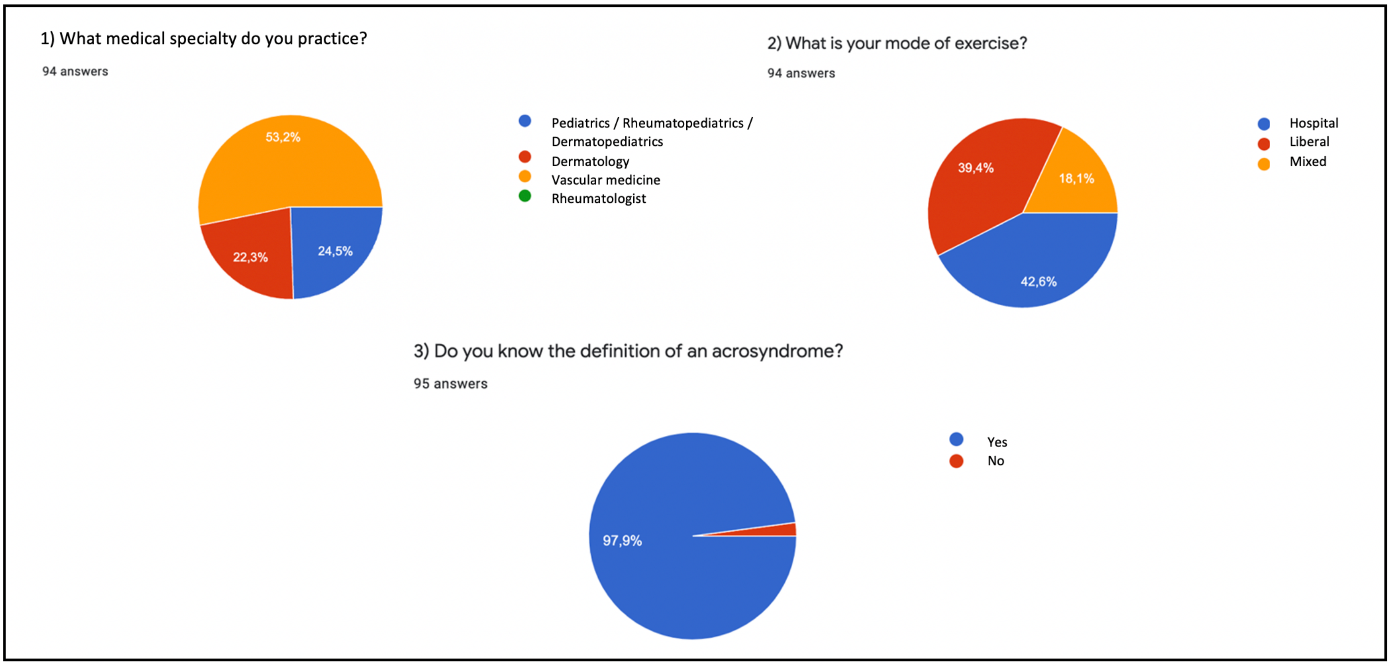

Part 1: acrosyndromes in children

4) Have you ever had children with an acrosyndrome among your patients?

95 answers

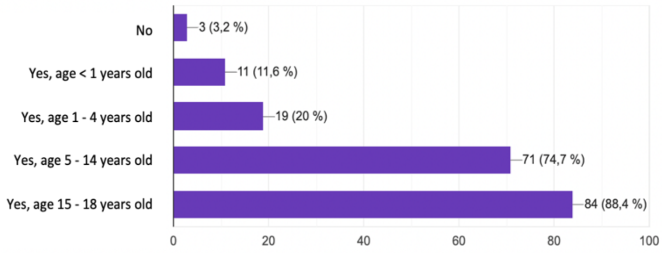

5) If yes, which one(s)?

92 answers

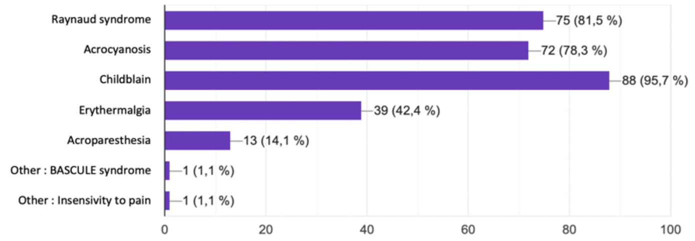

6) What is the proportion?

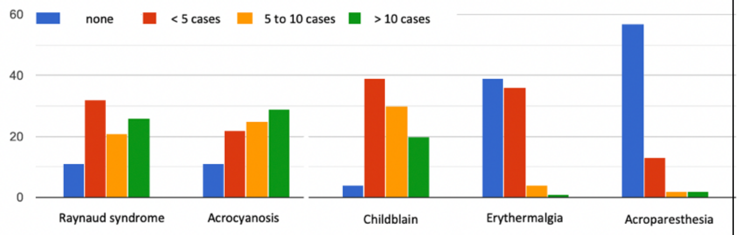

7) Can you quantify the total number of cases of acrosyndromes encountered in children over the past 10 years?

95 answers

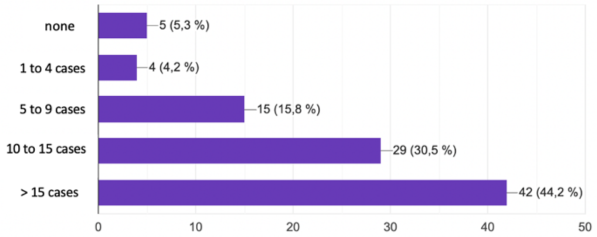

8) During which season are the most patients consulting you for this problem?

88 answers

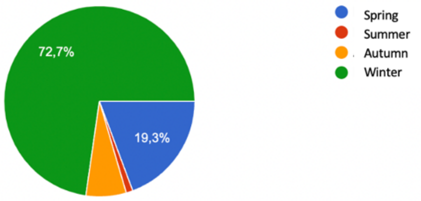

9) Faced with a child's acrosyndrome (ie < 18 years old), do you systematically carry out additional examinations?

92 answers

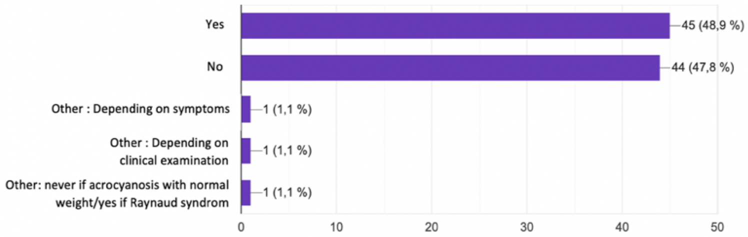

10) What additional examinations have you already prescribed?

88 answers

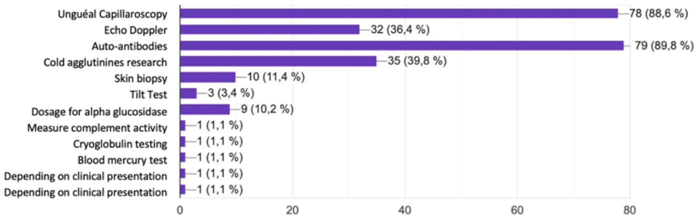

11) In the context of an acrosyndrome, have you found any family history?

93 answers

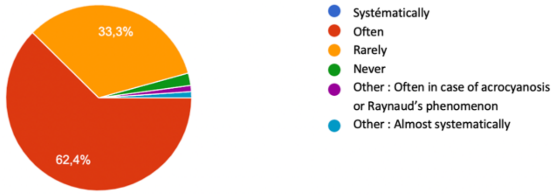

12) Depending on your specialty, to whom do you refer these patients?

92 answers

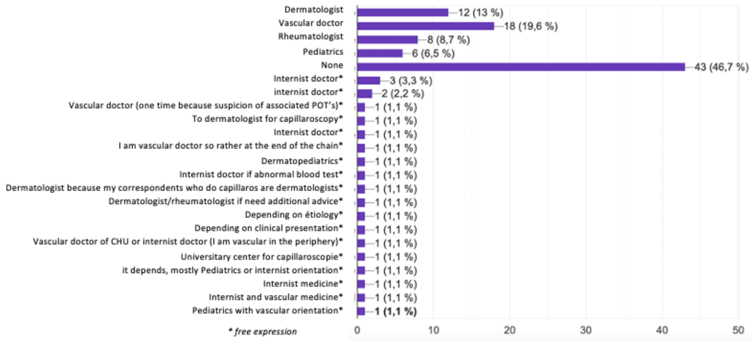

13) What treatments do you prescribe?

92 answers

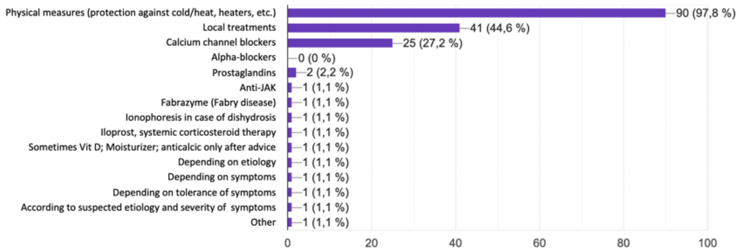

Part 2 : The "BASCULE" syndrome

14) Do you know the « BASCULE » syndrome

93 answers

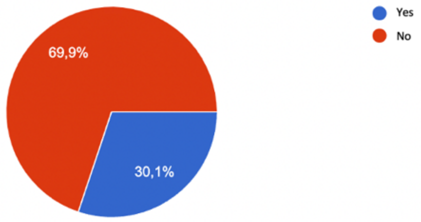

15) Have you ever encountered this type of lesion?

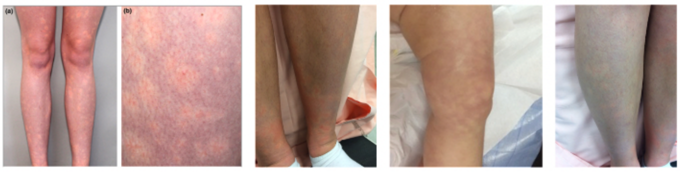

93 answers

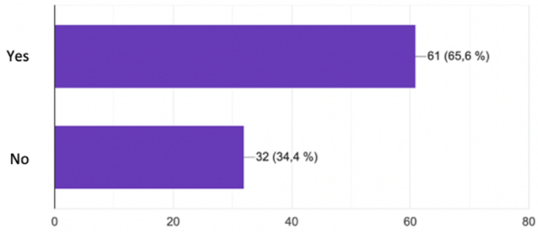

16) In what age group did you observe them?

87 answers

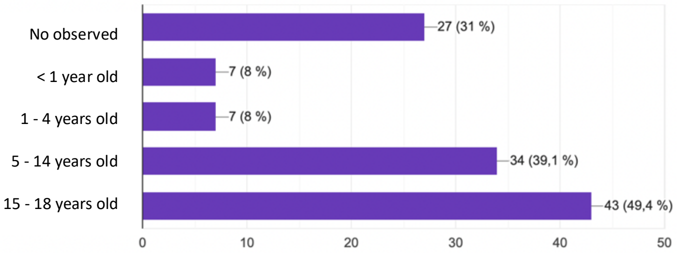

17) If yes, how many cases have you encountered?

62 answers

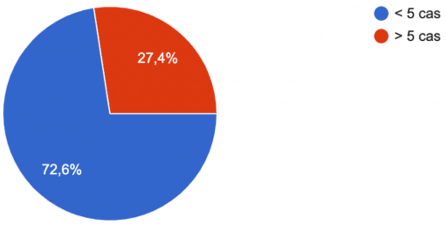

18) Faced with this syndrome, what additional examinations did you request?

59 answers

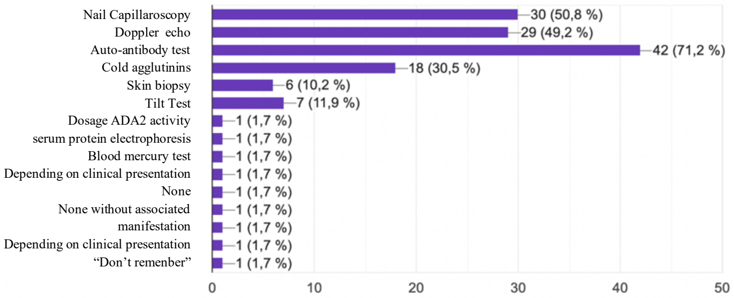

19) What treatments did you prescribe?

55 answers

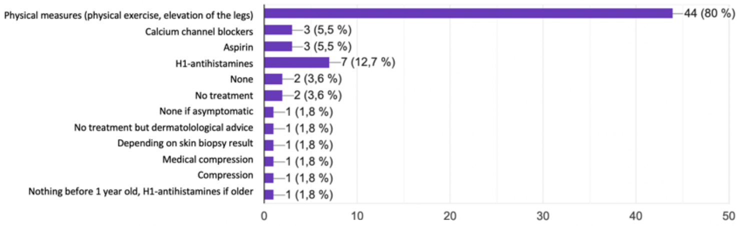

20) What was the evolution?

54 answers

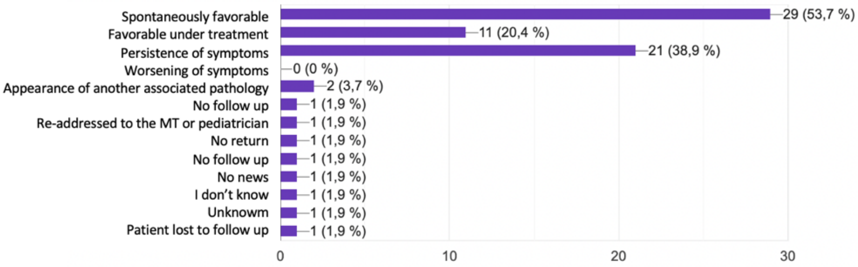

Supplement: Supplementary file 1 [file Data_Sheet_1.PDF]
